# Effects of Traditional Chinese Herbal Feed Additive on Production Performance, Egg Quality, Antioxidant Capacity, Immunity and Intestinal Health of Laying Hens

**DOI:** 10.3390/ani13152510

**Published:** 2023-08-03

**Authors:** Baiheng Liu, Ruyue Ma, Qinlin Yang, You Yang, Yuanjing Fang, Zhihong Sun, Daijun Song

**Affiliations:** 1 Key Laboratory for Bio-Feed and Animal Nutrition, College of Animal Science and Technology, Southwest University, Chongqing 400715, China; a251211455@163.com (B.L.); 13883137707@163.com (R.M.); 13399820021@163.com (Q.Y.); youygz@163.com (Y.Y.); 18250600712@163.com (Y.F.); 2 Chongqing Institute of Medicinal Plant Cultivation, Chongqing 408435, China

**Keywords:** traditional Chinese herbal feed additive, laying hens, production performance, egg quality, antioxidant capacity, immunity, intestinal health

## Abstract

**Simple Summary:**

Dietary supplementation with Chinese herbal feed-additives benefits the high production performance of laying hens. Considering that the effects of the Chinese herbal formulation on animal production differ, appropriate formulation may have additive effects. Therefore, this study investigated effects of the traditional Chinese herbal feed-additive prepared from specific Chinese herbs on production performance, egg quality, antioxidant capacity, immunity, and intestinal health of laying hens. The results of this study indicate that dietary supplementation with the traditional Chinese herbal feed-additive can effectively improve production performance, egg quality, antioxidant capacity, immunity, and intestinal health. The results revealed that the traditional Chinese herbal feed additive is an effective alternative to antibiotics in an antibiotic-free farming environment.

**Abstract:**

Chinese herbs have been used as feed additives in animal production. This study investigated the effects of a Chinese herbal feed-additive (TCM, which contained *Elsholtzia ciliate*, *Atractylodes macrocephala*, *Punica granatum pericarpium*, and *Cyperus rotundus*) on the production performance, egg quality, antioxidant capacity, immunity, and intestinal health of Roman laying hens. A total of 720 28-week-old hens were randomly allotted to three groups with six replicates of forty hens each. The groups were fed a basal diet (CON group), a basal diet with 50 mg/kg zinc bacitracin (ABX group), or a basal diet with 400 mg/kg TCM (TCM group) for 56 days. The results showed that the TCM group increased egg production, egg mass, albumen height, and Haugh unit compared with the CON group (*p* < 0.05). There were no significant differences in egg weight, feed intake, feed conversion rate, and eggshell strength among all three groups (*p* > 0.05). Compared with the CON group, the TCM group enhanced the activities of glutathione peroxidase, total antioxidant capacity, and superoxide dismutase in serum and liver, and reduced malondialdehyde content (*p* < 0.05). The TCM also increased the levels of interleukin-2, interferon-γ, immunoglobulin A, immunoglobulin M, and immunoglobulin G, and decreased the levels of interleukin-6 and interleukin-8 compared with the CON group (*p* < 0.05). Furthermore, the TCM group increased jejunal goblet cell density and decreased ileal crypt depth and lymphocyte density compared with the CON group (*p* < 0.05). The results of 16S rRNA demonstrated that the TCM can change the diversity and composition of intestinal microbiota. At the phylum level, the abundance of *Bacteroides* increased while that of *Firmicutes* decreased in the TCM group (*p* > 0.05). At the genus level, the abundance of *Lactobacillus*, *Rikenellaceae_RC9_gut_group*, and *Phascolarctobacterium* increased while that of *Bacteroides* and *unclassified_o__Bacteroidales* decreased in the TCM group (*p* > 0.05). The effects of ABX were weaker than those of the TCM. In conclusion, the TCM has positive effects on production performance and the intestinal health of hens.

## 1. Introduction

Antibiotics have been widely used in poultry production for decades to prevent diseases and promote growth. However, the continuous use of antibiotics has led to increased drug resistance in pathogens, accumulation of antibiotic residues in animal products and the environment, and imbalance of normal microbial communities, causing serious harm to humans, animals, and the environment [1]. In 2006, European countries restricted or banned the use of antibiotics as growth promoters in response to antibiotic resistance and abuse. In 2017, the U.S. Food and Drug Administration (FDA) banned the use of antibiotics as feed supplements to promote growth in livestock and poultry. In July 2020, China also passed legislation to ban the use of antibiotics in farming [2]. Therefore, it is necessary for the poultry industry to develop alternative growth promoters to achieve “antibiotic-free” animal farming.

In poultry production, feed additives prepared from different Chinese herbs have received increasing attention as alternatives to antibiotics, anti-inflammatories, antioxidants, and immune stimulants to regulate intestinal microbiota and promote growth [3]. The beneficial effects of Chinese herbs on poultry production and health come from their richness in polysaccharides, polyphenols, and other active ingredients that can improve metabolism and prevent animal diseases [4]. The addition of Chinese herbs to the diets of weaned piglets improve antioxidant capacity and affect the abundance of *Firmicutes* and *Bacteroidota* in the intestinal microbiota [5]. Bai and Li [6] added Chinese herbs to the diets of laying hens and significantly improved production performance, egg quality, nutrient retention, and immunity.

Chinese herbs have been used in the medical field in China for thousands of years [7]. *Elsholtzia ciliate*, *Atractylodes macrocephala*, *Punica granatum pericarpium*, and *Cyperus rotundus* are commonly used traditional Chinese herbs with various health benefits. Their safety has been recognized by numerous researchers and they are widely used in livestock and poultry breeding [7,8,9,10]. These herbs contain a variety of active ingredients, including organic acids, polyphenols, and polysaccharides, that have been reported to have positive effects on livestock production [8,9,10]. However, the effects of combinations of Chinese herbs on laying hens may vary depending on the specific combination used. A herbal mixture composed of several herbs contains multiple active ingredients that may exhibit greater biological efficiency than a single herbal extract [11]. To date, the combination of *Elsholtzia ciliate*, *Atractylodes macrocephala*, *Punica granatum pericarpium* and *Cyperus rotundus* has not been extensively studied in laying hens. Therefore, based on long-term practical experience and a theoretical summary of traditional Chinese medicine, we use natural properties, flavors, and interrelationships of natural herbs as the main basis for making natural, efficient, and harmless practical Chinese herbal feed-additive (TCM) with *Elsholtzia ciliate*, *Atractylodes macrocephala*, *Punica granatum pericarpium* and *Cyperus rotundus* as the main ingredients. The TCM is added to the diet of laying hens to further explore its effects on egg production, egg quality, antioxidant properties, immune performance, intestinal histomorphology, and the cecal microbiota of laying hens, in order to provide new ideas for the development of antibiotic-free breeding technology for animals.

## 2. Materials and Methods

### 2.1. The TCM and Antibiotics

The TCM used in this study was provided by Caomu Jinhua Technology Co., Ltd. located in Chengdu, China. The TCM formulation consisted of a combination of 8% *Elsholtzia ciliata*, 3% *Atractylodes macrocephala*, 7% *Punica granatum pericarp*, and 7% *Cyperus rotundus*, and its major active ingredients were tannins, polysaccharides, flavonoids, saponins, tannic acids, etc., with 75% soybean shell powder as the carrier. The concentrations of polysaccharides, flavonoids, and polyphenols in the TCM were ≥7%, ≥2%, and ≥6.5% (as dry weight), respectively, and the combined concentration of organic acids and saponins was approximately 10%. The total number of mold was <4.0 × 10^4^ CFU/g, and ethylparaben was not detected in the TCM. The zinc bacitracin used in this study was sourced from XIN-XING, a veterinary pharmaceutical factory located in Tianjin, China.

### 2.2. Animals and Experimental Design

All experimental procedures involving laying hens were approved by the License of Experimental Animals (IACUC-20230609-07) of the Animal Experimentation Ethics Committee of Southwest University, Chongqing, China. A total of 720 Roman laying hens, 28 weeks old, were randomly assigned to one of three dietary treatments with six replicates of forty hens per replicate. The experiment lasted for 8 weeks (week 28–36 of the hens’ age), during which the three groups were fed different diets: a basal diet (CON group), a basal diet supplemented with 50 mg/kg zinc bacitracin (ABX group), and a basal diet supplemented with 400 mg/kg TCM (TCM group). The ingredients and chemical composition of the basal diet are presented in Table 1. Hens were kept in an environmentally controlled house with free access to water and mash feed in 3-level cages with controlled ventilation and lighting (light duration: 16 h; light intensity: 8 lx). Eggs were collected and weighed at the same time every afternoon. 

### 2.3. Sample Collection

On day 56, 12 laying hens were randomly selected from each group (2 per replicate). After fasting for 12 h, blood samples (10 mL) were obtained from the wing vein and centrifuged at 3000× *g* at 4 °C for 10 min. The serum was stored at −80 °C for further analysis. The hens were then euthanized by cervical dislocation and liver tissues were removed, washed, packed, and frozen for later analysis of oxidation status. Two segments each of the jejunum and ileum were excised and stored in formalin solution for histological examination. The digesta samples of the cecum, which were approximately 5 mL, were collected aseptically and stored at −80 °C for analysis of 16S RNA.

### 2.4. Production Performance and Egg Quality

The number of eggs, egg weight, feed consumption, and mortality were recorded every day. The egg production and average egg weight were calculated based on the total egg numbers and total egg weight during the entire experimental period. The egg mass was calculated by multiplying the total egg weight by the egg production. The feed conversion ratio (FCR) was calculated by dividing the feed intake by the egg mass. To determine egg quality, 60 eggs were randomly collected per treatment group (10 eggs per replicate). Each egg was individually weighed and broken using an egg force reader (Orka Food Technology, Bountiful, UT, USA) to measure eggshell breaking strength. The eggshell thickness was measured using a micrometer screw gauge (Suce measuring instrument Co., Ltd., Nanjing, Jiangsu, China) after removing the shell membrane and is represented as the average thickness of the upper, middle, and lower end of the shell. The albumen height and Haugh unit were analyzed using an egg quality tester (Orka Food Technology, Bountiful, UT, USA).

### 2.5. Assay of Antioxidant Indices in the Serum and Liver

To assess the activity of antioxidant enzymes, the serum and liver were analyzed for superoxide dismutase (SOD), glutathione peroxidase (GSH-PX), total antioxidant capacity (T-AOC), and content of malondialdehyde (MDA). Enzymatic activities were measured using an assay kit obtained from Nanjing Jiancheng Bioengineering Institute, and the procedures were carried out strictly according to the manufacturer’s protocol. The intra- and inter-assay CVs for these kits were both less than 10.0%.

### 2.6. Serum Immunologic Indices

The levels of immunoglobulins (IgA, IgG, IgM), interleukin-2 (IL-2), interleukin-6 (IL-6), interleukin-8 (IL-8), gamma-interferon (IFN-γ), and tumor necrosis factor-α (TNF-α) in the serum were determined by ELISA with enzyme marker. The commercial kits were sourced from Chongqing Pengguang Biotechnology Co., Ltd. (Chongqing, China). The intra- and inter-assay CVs for these kits were both less than 10.0%.

### 2.7. Intestinal Morphology

After sacrificing the laying hens on day 56, the jejunum and ileum of laying hens were collected. Next, 2 cm segments were removed from the mid-jejunum and mid-ileum from each laying hen and fixed in a 10% neutral buffered formalin for more than a week to make intestinal sections. All intestinal segments from each jejunum and ileum were embedded after removing the fixation solution, using standard paraffin embedding procedures. Subsequently, a 5-μm section of each sample was located on a glass slide and stained by hematoxylin and eosin for light microscopy. A light microscope was used to determine the histological indices, including villus height, crypt depth, goblet cell density, and lymphocyte density [3].

### 2.8. Bacteria 16S rRNA Sequencing and Bioinformatics Analysis

The experiment was conducted based on the methods of previous researchers [5,12]. Total DNA from the cecal digesta was extracted using the EZNAVR DNA Isolation Kit (Omega Biotek, Norcross, GA, USA) following the manufacturer’s instructions. The hypervariable regions (V3 and V4) of the 16S RNA gene were amplified using primers 338F (′ACTCCTACGGGAGGCAGCA-3′) and 806R (5ʹGGACTACHVGGGTWTCTAAT-3′). Amplicons were run on 2% agarose gels and bright main bands of 400–450 bp were selected for analysis. The amplified products were purified with an AxyPrep DNA Gel Extraction Kit (Axygen Biosciences, Union City, CA, USA) and sequences were determined on the Illumina MiSeq platform (Illumina, San Diego, CA, USA) following the instructions from Majorbio BioPharm Technology Co., Ltd. (Shanghai, China).

Raw fastq files were quality-filtered using Trimmomatic and merged using FLASH 1.2.1. UPARSE was used to identify operational taxonomic units (OTUs) created by clustering at 97% sequence similarity and removing chimeric sequences. Species classification was annotated using the Silva database (release 138). Statistical analysis was performed using the Majorbio Cloud Platform. Alpha diversity (Sobs, Shannon, Simpson, and Bootstrap indices) was analyzed based on the OTU data [13]. Partial least squares discriminant analysis (PLS−DA) was performed to compare microbial composition.

### 2.9. Statistical Analysis

One-way analysis of variance was used for statistical analysis using SPSS 25.0 soft-ware. Any differences among treatments were then compared using the Duncan comparison range tests. The experimental data are expressed as the means ± SEM; *p* < 0.05 among different groups was considered statistically significant.

## 3. Results

### 3.1. Production Performance

As shown in Table 2, compared with the CON group, the results showed that the TCM group increased egg production and egg mass (*p* < 0.05) during the trial period, but had no significant effect on egg weight, feed intake, and FCR (*p* > 0.05). In addition, the ABX group had no significant effect on production performance compared with the CON group.

### 3.2. Egg Quality

As shown in Table 3, compared with the CON group, the results showed that the ABX and TCM groups had a significant influence on albumen height and Haugh unit (*p* < 0.05) and had no significant influence on eggshell strength (*p* > 0.05). In addition, the eggshell thickness was numerically higher in the ABX group than the TCM group (*p* < 0.05).

### 3.3. Antioxidant Capacity

As shown in Table 4, compared with the CON group, the TCM group increased the activities of GSH-Px, SOD, and T-AOC in the liver and serum (*p* < 0.05). Additionally, the content of MDA decreased in the TCM group (*p* < 0.05). Compared with the CON group, the ABX group had no significant effect on the liver and serum antioxidant indices (*p* > 0.05) except for liver MDA.

### 3.4. Immunity

As shown in Table 5, compared with the CON group, the TCM group had higher levels of serum IL-2, IFN-γ, IgA, IgM, and IgG (*p* < 0.01), and lower levels of serum IL-6 and IL-8 (*p* < 0.05). Moreover, the TCM group demonstrated a more significant effect on the aforementioned indices compared with the ABX group.

### 3.5. Intestinal Morphology

**Figure 1:**
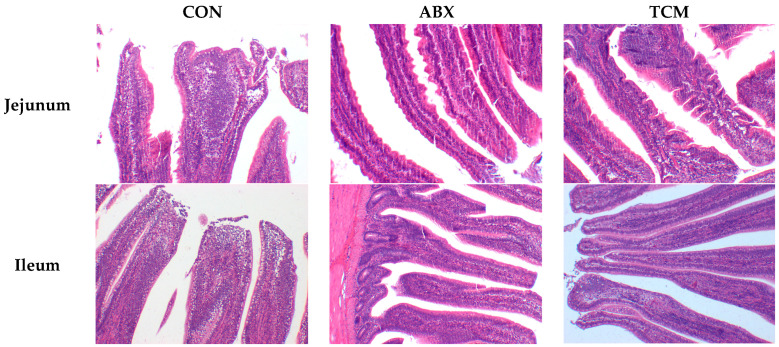
Effects of supplementing the diets with different combinations of the TCM and ABX on the intestinal (jejunum, and ileum) morphology in laying hens (100×).

The light micrographs in Figure 1 show exfoliation of a few cells from the tip of jejunal villi in the CON and ABX groups, while the jejunal villi in the TCM group appeared more intact. The integrity of ileal villi in the CON group was lower compared to other groups. These findings were further supported by the data in Table 6, which showed that the ABX group increased jejunal villus height (*p* < 0.05) and the TCM group increased jejunal goblet cell density (*p* < 0.05) compared with the CON group. In the ileum, the TCM and ABX groups decreased ileal crypt depth (*p* < 0.05) and increased ileal lymphocyte density (*p* < 0.05). In addition, the ABX group decreased ileal goblet cell density (*p* < 0.05).

### 3.6. Microbial Composition

To identify the microbial composition in the cecal digesta of Roman laying hens, 16s RNA MiSeq sequencing was performed. The results revealed that dietary supplementation with the TCM resulted in improved production performance indices.

**Figure 2:**
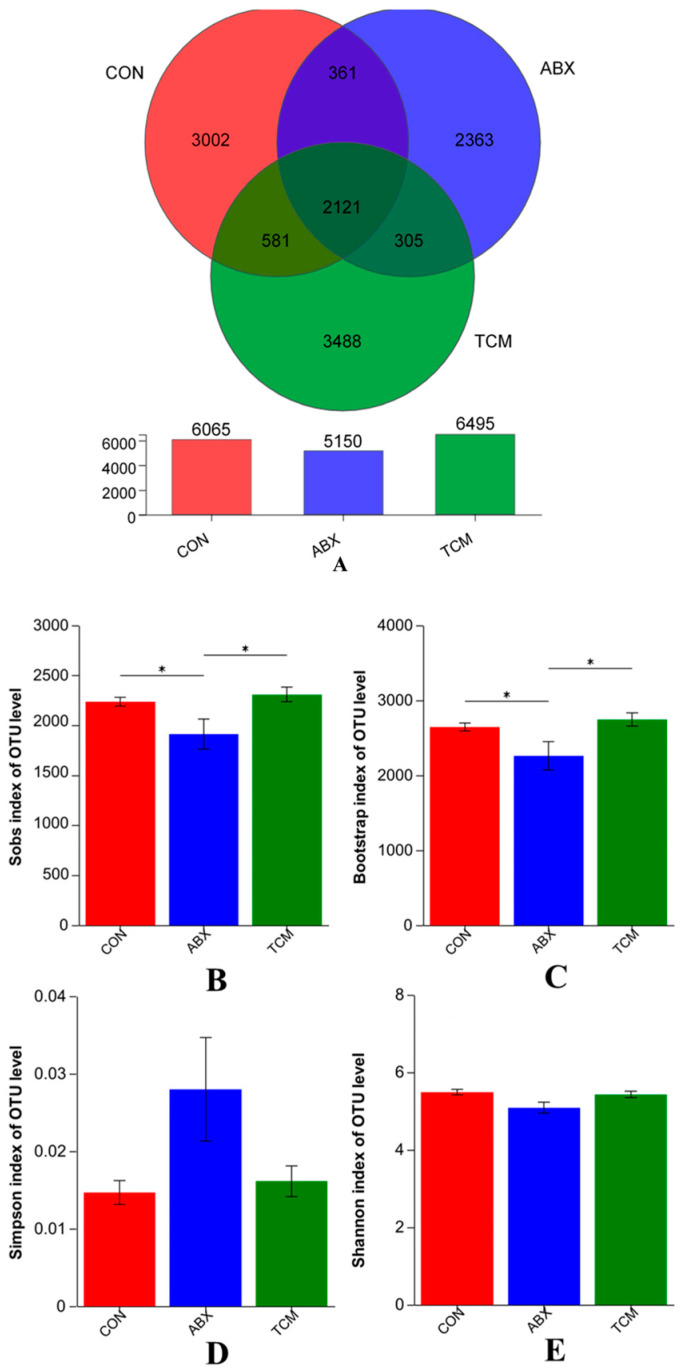
Effect of the TCM on the number of intestinal OUT and alpha diversity of laying hens. (**A**), number of intestinal OUT of laying hens; (**B**), Sobs index; (**C**), Bootstrap index; (**D**), Shannon index; (**E**), Simpson index. *n* = 5. * represents *p* < 0.05.

**Figure 3:**
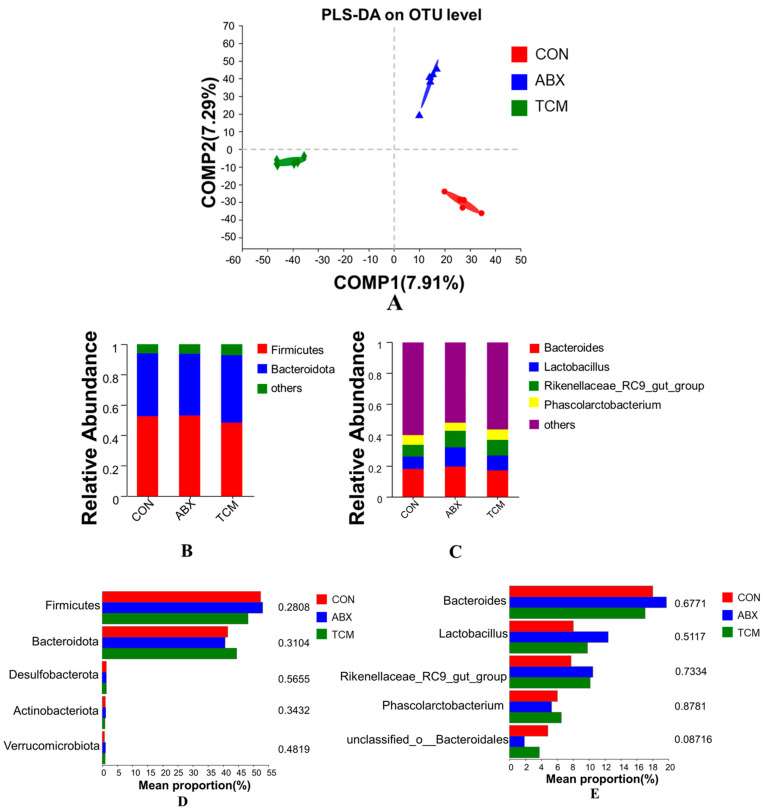
Analysis of the composition of cecal microbiota. (**A**), PLS−DA on OTU level; (**B**), Barplot analysis of microbial community compositions at phylum levels; (**C**), Barplot analysis of microbial community compositions at genus levels; (**D**), Test of difference between groups at phylum levels; (**E**), Test of difference between groups at genus levels. *n* = 5.

All samples were clustered into OTUs with 97% identity. The 2121 shared OTUs were detected in all groups, and the number of unique OTUs for the CON, ABX, and TCM groups were 3002, 236,3 and 3488, respectively (Figure 2A). The alpha diversity indices of intestinal microbiota were presented in Figure 2B–E, with the TCM group having the highest Sobs and Bootstrap index among the three groups, while the ABX group had the opposite. Lower Shannon indices and higher Simpson indices were observed in the ABX group compared to the CON and TCM groups, but there was no significant difference in Shannon and Simpson indices between the CON group and the TCM group. To evaluate the extent of similarity of the bacterial communities, PLS−DA at the OTU level was employed and indicated obvious separation between groups (Figure 3A). PLS−DA also showed that the bacterial communities of the TCM group clustered in the third quadrant, while ABX and CON groups clustered in the first and fourth quadrants, respectively, suggesting that the overall structures of the bacterial communities in the groups were different. The microbial composition and abundance in the intestine at the phylum level are shown in Figure 3B,D, with *Firmicutes* and *Bacteroidota* being the dominant bacterial phyla. These two bacterial phyla accounted for 93.93%, 93.7%, and 92.64% of the entire microbiota in the CON, ABX, and TCM groups, respectively. Compared with the CON group, the ABX group had a tendency to increase the abundance of *Firmicutes* and decrease the abundance of *Bacteroides* (*p* > 0.05). In contrast, the abundance of *Firmicutes* decreased and *Bacteroides* increased in the TCM group (*p* > 0.05). At the genus level, intestinal microbiota was mainly composed of *Bacteroides*, *Lactobacillus*, *Rikenellaceae_RC9_gut_group*, *Phascolarctobacterium* and *unclassified_o__Bacteroidales* (Figure 3C,E). Compared with the CON group, the abundance of *Bacteroides* and *unclassified_o__Bacteroidales* in the TCM group was decreased (*p* > 0.05), while the abundance of *Lactobacillus*, *Rikenellaceae_RC9_gut_group* and *Phascolarctobacterium* was increased (*p* > 0.05). In the ABX group, the abundances of *Phascolarctobacterium* and *unclassified_o__Bacteroidales* were decreased (*p* > 0.05), while the abundances of *Bacteroides*, *Lactobacillus* and *Rikenellaceae_RC9_gut_group* were increased (*p* > 0.05). 

## 4. Discussion

Numerous studies have demonstrated that dietary supplementation with Chinese herbs effectively improves the production performance of laying hens. Dilawar et al. [14] reported a significant increase in egg production, egg weight, and egg mass in laying hens fed diets containing *Mentha arvensis* and *Geranium thunbergii*. In the present study, dietary supplementation with the TCM significantly increased the egg production and egg mass of laying hens. Additionally, these results regarding the improvement in intestinal morphology by the TCM support this notion. Intestinal morphology is a useful marker for estimating the digestion and absorption capacity of animals [15]. A lower intestinal villus height or deeper crypt indicates decreased nutrient absorption ability in the small intestine [16,17]. This study demonstrated that dietary supplementation with the TCM had a positive effect on intestinal morphology, which is consistent with the studies of Wang et al. [17]. It may be attributed to the rich content of active ingredients in the TCM such as esters, unsaturated fatty acids, and sugars, which provide sufficient nutrients for the growth of intestinal villi epithelial cells [9,18]. Moreover, the density of small intestinal lymphocytes and goblet cells, which are associated with intestinal immunity, was significantly increased in the TCM group. It is suggested that the TCM may improve immunity by improving intestinal morphology, ultimately leading to an improvement in production performance. However, some studies have shown that Chinese herbs failed to impact production performance and intestinal morphology [19]. This variation in efficacy may be attributed to differences in the formulations and dosages of Chinese herbs used [11,15].

The quality of eggs can be improved by controlling the diet of laying hens [20]. Early research showed that dietary supplementation with Chinese herbs (a mixture of *R. astragali*, *S. miltiorrhiza Bunge*, and *C. monnieri*) significantly improved the albumen height and Haugh unit of eggs [21]. In this study, albumen height and Haugh unit were greater in laying hens fed the TCM diet compared with laying hens fed the basic diets. Plant-derived polyphenols and polysaccharide extracts exhibit potent antioxidant activity, which can minimize protein degradation by reducing lipid and protein oxidation [22,23]. The TCM contains polysaccharides and polyphenols from a variety of Chinese herbs, and their combination may account for the improvement in egg quality in this study. However, some researchers have suggested that Chinese herbs have little effect on egg quantity [19,24]. Variations in the age of selected hens may account for these differences, as aging is an important factor affecting egg quantity in newly laid eggs [22,25,26].

Excessive production of free radicals in laying hens can lead to oxidative damage, resulting in cellular, DNA, protein, and lipid damage, ultimately affecting poultry production and health [27,28]. SOD and GSH-Px are the first line of enzymatic antioxidant defense, and serve as specific scavengers of free radicals, making them markers of activated antioxidant enzyme systems [4]. T-AOC is used to assess the overall antioxidant capacity of an organism [29]. MDA is the primary product of lipid peroxidation and is commonly used as a biomarker to assess oxidative damage [4,30]. In the present study, the TCM group increased the activities of GSH-Px, T-AOC, and SOD and reduced the content of MDA in both the liver and serum of laying hens, consistent with previous studies [30,31]. Xu et al. [30] showed that supplementation of a Chinese herbal mixture in weaned pigs’ diets significantly increased the activities of T-AOC and decreased the content of MDA in the serum. Similarly, Long et al. [31] observed that the supplementation of *Lycium barbarum* polysaccharide in broiler diets increased the activity of SOD and decreased the content of MDA in the chicken liver. The results indicate that the TCM can increase the antioxidant capacity of laying hens by increasing the activities of antioxidant enzymes. This further confirms the notion that the TCM improves egg quality by enhancing antioxidant capacity, as mentioned earlier. In addition, the TCM exhibits a stronger effect on antioxidant indices than ABX, indicating that it can be used as an alternative to antibiotics.

Active ingredients, such as the polysaccharides and polyphenols contained in Chinese herbs, are believed to enhance poultry immunity by regulating the levels of immunoglobulins and cytokines in poultry [4,9,32]. IFN-γ, an essential index cytokine, plays a key role in the cell-mediated immune response [33]. IL-2, a cytokine with broad-spectrum immune promoter activity, is crucial for cellular and humoral immunity and plays a significant role in numerous cytokine networks [18]. IgA, IgM, and IgG are the primary serum immunoglobulins, and their levels in the serum are closely associated with the immunity of laying hens [34]. In this study, the TCM group showed increased levels of IL-2, IFN-γ, IgA, IgM, and IgG and decreased levels of the pro-inflammatory cytokines IL-6 and IL-8. These findings suggest that the TCM could enhance the immunity of laying hens and suppress the inflammatory response. Xu et al. [35] also reported similar results, demonstrating that appropriate doses of Chinese herbal mixture could be used as novel and effective immunostimulants for livestock. The intestine is widely recognized as the largest immune organ in animals. It contains many immunoreactive lymphocytes that protect against bacterial and viral invasion by influencing the secretion of cytokines and antibody molecules such as IL-2 and IFN-γ [36,37,38]. Therefore, it can be hypothesized that the elevated levels of immune molecules such as IL-2, IFN-γ, IgA, IgM, and IgG in the serum could be due to the proliferation of small intestinal lymphocytes stimulated by the TCM, which is consistent with the increased density of ileal lymphocytes observed in this study [37,38,39]. In line with this study, Li et al. [39] and Qin et al. [32] also demonstrated that Chinese herbs have immune-enhancing properties through the promotion of intestinal lymphocyte proliferation. Moreover, there is a strong correlation between the antioxidant system and the immune system. Reactive oxygen species (ROS) play a significant role in a variety of diseases, including chronic inflammation. Excessive production of ROS leads to oxidative stress, which is a crucial factor contributing to immune deficiency [40]. This indicates that the TCM-mediated antioxidant capacity could also be responsible for its immune-enhancing capacity. In conclusion, this study suggests that the TCM may modulate the immune system through mechanisms involving ROS scavenging and production, cytokine secretion, and immune cell proliferation [32,35,39,41]. 

The intestine of laying hens contains a complex microbiota, whose composition and abundance have been proven to be closely associated with the production and health of laying hens [42,43,44]. Previous studies have indicated that the active ingredients in Chinese herbs can influence the composition of intestinal microbiota [45,46]. The Sobs and Bootstrap indices are commonly used to assess microbial richness, while the Shannon and Simpson indices are used to evaluate microbial diversity [47]. Changes in these indices suggest that the TCM had a positive effect on the richness and diversity of cecal microbiota, while ABX had a negative effect. PLS−DA analysis revealed that the TCM and ABX altered the structure of the cecal microbial community. This may be due to ABX inhibiting nutrient absorption by certain intestinal microbiota and slowing down the maturation of chicken intestinal microbiota. In contrast, plant polysaccharides in the TCM can be metabolized by microorganisms as an energy source to promote the growth and reproduction of intestinal microbiota [48,49].

Changes in the structure of intestinal microbiota may affect the normal digestive process of the diet. *Firmicutes* and *Bacteroidota* predominate in the cecal digesta of laying hens, consistent with previous studies [50]. Researchers have reported that obese animals exhibit a higher ratio of *Firmicutes*/*Bacteroidota* compared to individuals with normal weight [35,51]. Obesity in laying hens leads to suppression of estrogen production and reduces egg production [21]. In the present study, dietary supplementation with the TCM decreased the ratio of *Firmicutes*/*Bacteroidota* in the cecal digesta of laying hens, potentially explaining the improvement in egg production [3,5,52]. At the genus level, *Lactobacillus*, *Rikenellaceae_RC9_gut_group*, and *Phascolarctobacterium* were identified as major microbiota and increased in abundance with the TCM supplementation [53]. *Lactobacillus* is a beneficial bacterium associated with intestinal health. It can improve the antioxidant capacity of animals by scavenging excess reactive oxygen radicals and increasing antioxidant enzyme activity through lactic acid production [54]. Lactic acid also can maintain the overall microbial structure by producing lactate to stimulate immune cell activity and competitively inhibiting pathogenic bacteria colonization in the intestine [55]. The abundance of *Lactobacillus* in the TCM and ABX groups was higher than the CON group, indicating that dietary supplementation with the TCM and ABX may increase the abundance of beneficial bacteria to promote the antioxidant capacity and immunity of laying hens. Short-chain fatty acids (SCFAs) play a crucial role in regulating intestinal microbiota, suppressing inflammation, and participating in nutrient metabolism [56,57]. Studies have shown that *Lactobacillus*, *Rikenellaceae_RC9_gut_group*, and *Phascolarctobacterium* can produce SCFAs, such as acetic acid, propionic acid, butyric acid, and succinic acid [58]. The increase in the relative abundance of *Lactobacillus*, *Rikenellaceae_RC9_gut_group*, and *Phascolarctobacterium* in the TCM group indicates an increase in SCFAs production, suggesting that the TCM might promote production performance by increasing SCFA-producing bacteria, but the specific mechanism of action needs to be studied in depth [55].

Currently, limited studies have investigated the effects of Chinese herbal mixtures on intestinal microbiota. The beneficial functions of Chinese herbal mixtures are attributed to their active ingredients such as polysaccharides, polyphenols, and organic acids [59,60]. In the present study, the TCM was found to be rich in polysaccharides, which have been shown to modify the composition and diversity of intestinal microbiota, including increasing the abundance of *Lactobacillus* in mice [45]. The TCM also contains high concentrations of polyphenols, which have been shown to regulate the intestinal microbial composition of animals and maintain intestinal health [60]. For instance, Wang et al. [17] demonstrated that tannic acid, a type of polyphenol, could alleviate obesity symptoms by reducing the ratio of *Firmicutes*/*Bacteroides* in weaned piglets. Furthermore, the TCM is a potential source of organic acids, which can lower the pH value in the gastrointestinal tract and inhibit the growth of some pathogenic bacteria, such as *Escherichia coli* [61]. Therefore, the changes in intestinal microbiota after dietary supplementation with the TCM could be attributed to the content of polysaccharides, polyphenols, or organic acids. Further studies are needed to understand the potential mechanisms underlying these effects.

## 5. Conclusions

This study demonstrates that supplementing the diet of laying hens with the Chinese herbal feed-additive containing *Elsholtzia ciliate*, *Atractylodes macrocephala*, *Punica granatum pericarpium* and *Cyperus rotundus* can improve production performance and egg quality. These improvements are associated with changes in the antioxidant capacity, immunity, intestinal morphology, and intestinal microbiota. Notably, the TCM appears to promote a more stable and well-run intestinal microecology compared to antibiotics, which may help prevent the disruption of microbial balance. In summary, these findings suggest that the Chinese herbal feed-additive is a good dietary additive for animals.

## Figures and Tables

**Table 1:** Dietary ingredients and chemical composition (%, as-fed basis).

Ingredients	Content	Composition	Content
Corn	63.15	Metabolic energy ^2^ (MJ/kg)	11.67
Wheat bran	4.74	Crude protein	16.00
Soybean	21.24	Calcium	3.23
Limestone	7.755	Total phosphorus	0.62
Bicalcium phosphate	0.4	Available phosphorus	0.32
Soybean oil	1.53	Methionine	0.38
Choline chloride	0.09	Lysine	0.85
NaHCO_3_	0.09	Methionine + Cysteine	0.72
Mineral premix	0.9	Isoleucine	0.52
Methionine	0.045	Threonine	0.54
Phytase	0.03	Tryptophan	0.17
Vitamin premix ^1^	0.03	Valine	0.68

^1^ The premix for per kilogram diet included: vitamin A, 12,000 IU; vitamin D3, 1500 IU; vitamin E, 25 IU; vitamin K3, 1.0 mg; vitamin B1, 1.6 mg; riboflavin, 5.0 mg; pantothenic acid, 15 mg; nicotinic acid, 20 mg; vitamin B6, 6.0 mg; biotin, 0.2 mg; folic acid, 0.5 mg; vitamin B12, 0.01 mg; choline, 500 mg; copper, 20 mg; iron, 90 mg; zinc, 80 mg; manganese, 80 mg; iodine, 0.45 mg; selenium, 0.2 mg. ^2^ Metabolic energy is calculated value and others are measured values.

**Table 2:** Effects of dietary supplementation with the TCM on performance production of laying hens.

Items	CON	ABX	TCM	SEM	*p*-Value
Egg production (%)	95.10 ^b^	95.16 ^b^	96.97 ^a^	0.321	0.015
Egg weight (g)	57.33	57.66	57.71	0.136	0.498
Feed intake (g/hen/d)	117.19	117.48	118.99	0.649	0.502
Egg mass (g/hen/d)	54.53 ^b^	54.87 ^ab^	55.96 ^a^	0.254	0.044
FCR (g feed/g egg)	2.15	2.14	2.13	0.014	0.800

CON, a basal diet; ABX, the basal diet plus a blend of 50 mg/kg zinc bacitracin; TCM, the basal diet plus a blend of 400 mg/kg TCM. Data are presented as the means ± SEM (*n* = 6). ^a,b^ Values within a row with different superscripts differ significantly at *p* < 0.05.

**Table 3:** Effects of dietary supplementation with the TCM on egg quality of laying hens.

Items	CON	ABX	TCM	SEM	*p*-Value
Eggshell strength (kgf)	5.36	5.21	5.35	0.042	0.301
Eggshell thickness (mm)	0.508 ^ab^	0.515 ^a^	0.499 ^b^	0.003	0.090
Albumen height (mm)	5.10 ^b^	5.41 ^a^	5.55 ^a^	0.066	0.007
Haugh Unit	70.23 ^b^	72.55 ^a^	72.93 ^a^	0.459	0.022

CON, a basal diet; ABX, the basal diet plus a blend of 50 mg/kg zinc bacitracin; TCM, the basal diet plus a blend of 400 mg/kg TCM. Data are presented as the means ± SEM (*n* = 6). ^a,b^ Values within a row with different superscripts differ significantly at *p* < 0.05.

**Table 4:** Effects of dietary supplementation with the TCM on antioxidant capacity of laying hens.

Items	CON	ABX	TCM	SEM	*p*-Value
Liver					
GSH-Px (U/mg)	283.73 ^b^	309.67 ^ab^	338.64 ^a^	9.196	0.045
T-AOC (mmol/g)	0.16 ^b^	0.18 ^ab^	0.22 ^a^	0.007	0.002
SOD (U/mg)	340.76 ^b^	356.69 ^b^	445.20 ^a^	14.57	0.003
MDA (nmol/mg)	2.27 ^a^	1.75 ^b^	1.59 ^b^	0.112	0.027
Serum					
GSH-Px (U/mL)	592.78 ^b^	635.99 ^ab^	739.16 ^a^	23.59	0.028
T-AOC (mM)	0.58 ^b^	0.55 ^b^	0.71 ^a^	0.027	0.024
SOD (U/mL)	58.94 ^b^	62.18 ^b^	81.39 ^a^	2.301	<0.001
MDA (nmol/mL)	5.55 ^a^	4.72 ^ab^	4.23 ^b^	0.221	0.043

CON, a basal diet; ABX, the basal diet plus a blend of 50 mg/kg zinc bacitracin; TCM, the basal diet plus a blend of 400 mg/kg TCM. Data are presented as the means ± SEM (*n* = 6). ^a,b^ Values within a row with different superscripts differ significantly at *p* < 0.05.

**Table 5:** Effects of dietary supplementation with the TCM on immunity in laying hens.

Items	CON	ABX	TCM	SEM	*p*-Value
IL-2 (pg/mL)	22.71 ^c^	28.62 ^b^	41.14 ^a^	1.605	<0.01
IL-6 (pg/mL)	6.57 ^a^	6.06 ^a^	5.42 ^b^	0.201	0.059
IL-8 (pg/mL)	24.40 ^a^	23.24 ^a^	20.05 ^b^	0.672	0.018
TNF-α (pg/mL)	23.89	22.73	21.42	0.454	0.080
IFN-γ (pg/mL)	17.35 ^b^	18.23 ^b^	23.34 ^a^	0.803	0.002
IgA (μg/mL)	52.12 ^b^	61.45 ^b^	77.94 ^a^	2.917	<0.01
IgM (μg/mL)	134.74 ^b^	152.25 ^b^	184.18 ^a^	7.043	0.01
IgG (μg/mL)	279.23 ^b^	303.39 ^b^	424.46 ^a^	17.96	<0.01

CON, a basal diet; ABX, the basal diet plus a blend of 50 mg/kg zinc bacitracin; TCM, the basal diet plus a blend of 400 mg/kg TCM. Data are presented as the means ± SEM (*n* = 6). ^a,b,c^ Values within a row with different superscripts differ significantly at *p* < 0.05.

**Table 6:** Effects of dietary supplementation with the TCM on intestinal morphology of laying hens.

Items	CON	ABX	TCM	SEM	*p*-Value
Jejunum					
Villus height (μm)	1042.37 ^b^	1242.45 ^a^	1042.68 ^b^	40.88	0.060
Crypt depth (μm)	98.10	101.14	86.82	5.05	0.504
V/C	11.52	12.67	12.29	0.80	0.846
Lymphocytes	88.67 ^ab^	74.67 ^b^	107.67 ^a^	6.57	0.061
Goblet cell	6.33 ^b^	8.00 ^b^	18.33 ^a^	2.02	0.002
Ileum					
Villus height (μm)	725.75	757.37	721.48	32.97	0.901
Crypt depth (μm)	121.28 ^a^	89.80 ^b^	87.00 ^b^	5.39	0.006
V/C	6.27	8.59	8.29	0.78	0.099
Lymphocytes	52.75 ^b^	86.5 ^a^	99.00 ^a^	6.57	0.001
Goblet cell	60.33 ^a^	33.33 ^b^	66.67 ^a^	5.46	0.002

CON, a basal diet; ABX, the basal diet plus a blend of 50 mg/kg zinc bacitracin; TCM, the basal diet plus a blend of 400 mg/kg TCM. Data are presented as the means ± SEM (*n* = 6). ^a,b^ Values within a row with different superscripts differ significantly at *p* < 0.05.

## Data Availability

The data presented in this study are available on request from the corresponding author.
